# The Health and Quality of Life Development Plan as a policy advisory tool for municipalities

**DOI:** 10.1093/eurpub/ckac129.474

**Published:** 2022-10-25

**Authors:** R Sequeira, P Pita Ferreira, S Carmezim Pereira, G Quinaz Romana, A Campos Fernandes, P Sousa

**Affiliations:** 1 National School of Public Health, NOVA University, Lisbon, Portugal; 2 Public Health Unit of Oeste Norte, ARSLVT, Caldas da Rainha, Portugal; 3 Public Health Unit António Luz, ARSLVT, Lisbon, Portugal; 4 Public Health Unit Francisco George, ARSLVT, Lisbon, Portugal; 5 Comprehensive Health Research Center, National School of Public Health, NOVA University, Lisbon, Portugal; 6 Public Health Research Centre, National School of Public Health, NOVA University, Lisbon, Portugal

## Abstract

**Issue:**

In 2019, the Portuguese government established the autonomy of local authorities and the decentralisation of public administration. Consequently, a process that includes the transfer of health competencies from the central government to the municipalities began. The newly acquired competencies enable local governments to play a crucial role in defining health policies and strategic partnerships with special focus on disease prevention, promotion of healthy lifestyles and active ageing. That said, several municipalities established a collaboration protocol with the National School of Public Health (NSPH) for the development of the Health and Quality of Life Development Plan (HQLDP) as a contribution to their strategic planning.

**Results:**

NSPH has developed a solidified methodology for municipalities to substantiate their health planning and priorities, based on a deep health diagnosis, from existing demographic, economical, health and environmental data in a given timeline. HQLDP is a medium-term reference that will support strategic action in the area of health and social determinants. The general objective of the HQLDP is to contribute to the improvement of the health status, in the different stages of the life cycle, based on the evaluation of the health profile and social determinants and to define a set of strategic objectives that promote the reduction of inequalities, the promotion and protection of health and the prevention of disease. The developed plan also includes public and stakeholders’ scrutiny, to define key areas of action in the health sector.

**Lessons:**

The HQLDP takes into account the vocation of local authorities to act on determinants through policies that intervene in the environmental, socio-economic, educational, urban planning and mobility contexts; assuming an active, influential and local role in health policies. Municipal intervention requires a strong and concerted local action and the definition of territorialized development strategies.

**Key messages:**

• HQLDP has a crucial role on the situational diagnosis and set of priorities of the quality of life and health indicators to assess the priorities at council level.

• HQLDP provides the knowledge for the development of public policies and interventions that comply with the population characteristics by reducing health inequalities and inequities.

